# The Eye Cannot See What the Brain Does Not Know: A Report of Two Cases of Morton’s Neuroma

**DOI:** 10.7759/cureus.103940

**Published:** 2026-02-19

**Authors:** Mehmet Oguz Yenidunya, Cagri Can Ilikci

**Affiliations:** 1 Plastic and Reconstructive Surgery, Bursa Uludağ University Faculty of Medicine, Bursa, TUR; 2 Medicine, Bursa Uludağ University Faculty of Medicine, Bursa, TUR

**Keywords:** interdigital nerve, metatarsalgia, morton's neuroma, neurectomy, plastic and reconstructive surgery

## Abstract

It is rare for patients to present to the plastic surgery department with foot pain. If this occurs, there is a high probability that these are patients who would have visited numerous hospitals without finding a solution to their complaints and have not recovered despite receiving various treatments. In such cases, Morton’s neuroma is a diagnosis that remains unseen by the eyes unless it is kept in mind. Following its correct diagnosis, surgical treatment is definitely within the scope of plastic surgery. In this report, we present two male patients, aged 35 and 40 years, treated in our clinic for chronic pain and burning sensation in the third intermetatarsal space. Both patients underwent surgical excision of the neuroma via a dorsal approach. Postoperatively, both patients reported significant improvement in their symptoms and returned to daily activities without complications. Although foot pain is primarily associated with orthopedics, Morton’s neuroma is an interdigital nerve pathology that requires precise surgical management. We conclude that plastic surgeons should not hesitate to diagnose and treat this condition, as it falls well within their area of expertise.

## Introduction

Plastic surgery practice encompasses a wide anatomical range extending from the scalp to the plantar region. Although foot-related conditions are not frequently encountered in routine plastic surgery practice compared to burns, congenital anomalies, skin tumors, and traumatic defects, patients with persistent symptoms despite prior evaluations may occasionally be referred to plastic surgery. In such situations, referral may reflect both diagnostic uncertainty and the expectation of comprehensive reconstructive expertise. Therefore, precise identification of the underlying pathology is essential.

Morton’s neuroma, also known as Morton’s metatarsalgia, is a compressive neuropathy affecting the common plantar digital nerve [1,2]. Despite its designation as a “neuroma,” it is not a true neoplasm but rather a benign perineural fibrosis characterized by degeneration and demyelination of nerve fibers [3]. The condition most commonly involves the third intermetatarsal space, followed by the second space [4]. It occurs more frequently in women, with an incidence rate of 87.5 per 100,000 compared to 50.2 per 100,000 in men [2]. Various etiological mechanisms have been proposed [5]. Clinically, patients typically report burning pain and paresthesia, often describing the sensation as “walking on a pebble.” Pain localized to the intermetatarsal space may also result from intermetatarsal bursitis, plantar plate injury, metatarsophalangeal joint synovitis, stress fractures of the adjacent metatarsal heads, Freiberg disease, ganglion cysts, or other soft tissue masses, all of which should be considered in the differential diagnosis.

Although forefoot pathology is frequently managed within orthopedic practice, Morton’s neuroma involves the digital nerves and requires meticulous nerve-oriented surgical technique. Given their expertise in peripheral nerve anatomy and surgical management, plastic surgeons are well-equipped to contribute to the evaluation and treatment of this condition.

## Case presentation

Case 1

A 35-year-old male patient presented with chronic pain and a burning sensation localized to the third intermetatarsal space of the right foot. The patient indicated a distinct point of tenderness upon deep palpation in this area. His medical history included a spontaneous pneumothorax. Prior to our evaluation, he had consulted the orthopedics and physical medicine and rehabilitation departments without experiencing symptom relief. Additionally, he had no occupational risk factors.

Clinical examination revealed tenderness localized to the third intermetatarsal space, with reproduction of symptoms upon deep palpation. Based on the typical localization of pain and consistent clinical findings, Morton’s neuroma was suspected. Radiological imaging was not performed. Given the characteristic localization of symptoms and reproducible tenderness on examination, the clinical findings were considered sufficiently suggestive of Morton’s neuroma.

**Figure 1:**
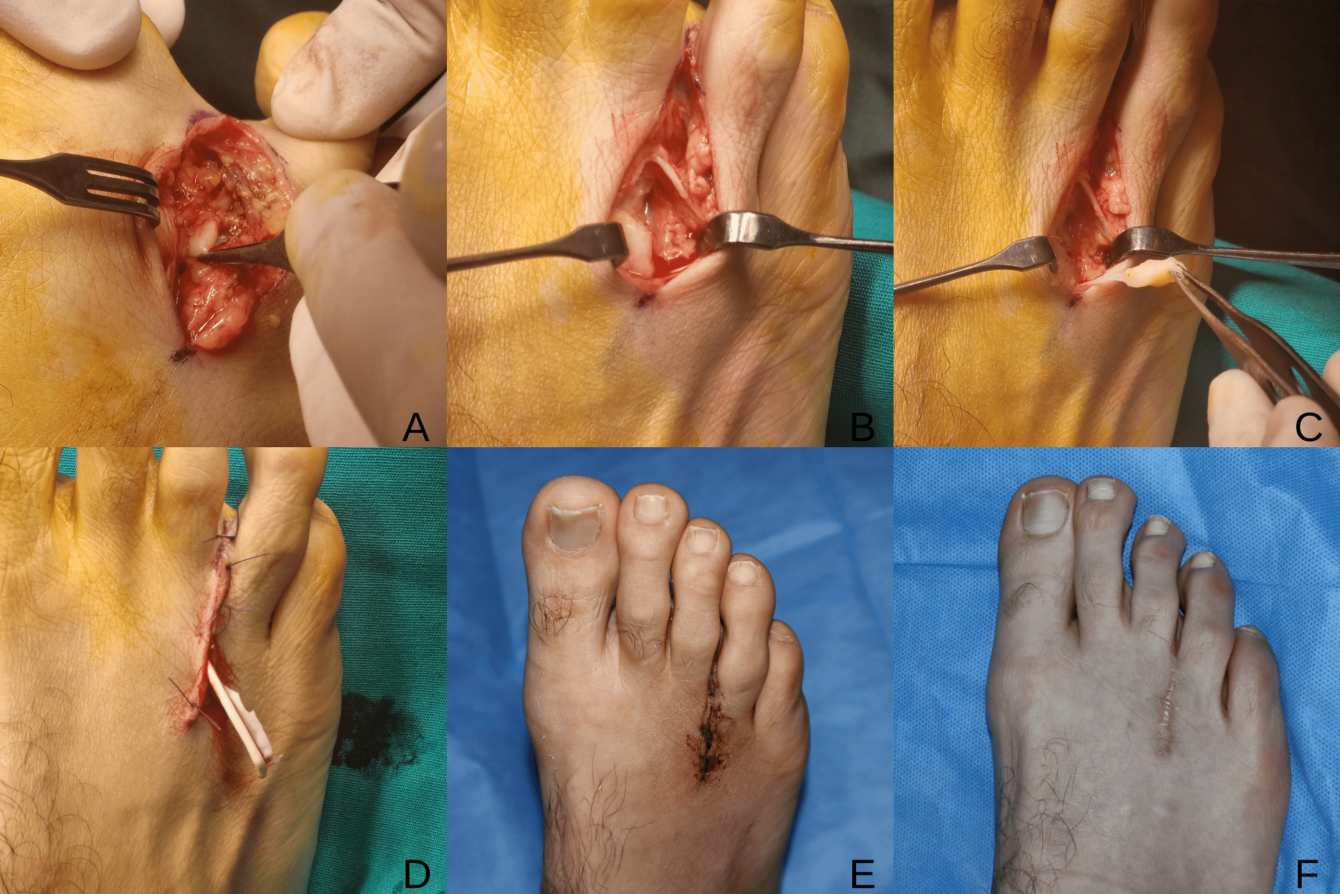
Intraoperative and postoperative views of case 1 (A,B): Dorsal longitudinal incision and exposure of the third intermetatarsal space. (C) Identification of Morton’s neuroma. (D) Wound closure with Penrose drain placement. (E) Early postoperative appearance. (F) Late postoperative scar at two-year follow-up.

The operation was performed under general anesthesia. Surgical excision of the neuroma was performed via a dorsal approach using a longitudinal incision. Sutures were removed on postoperative day 15. Postoperatively, the patient reported significant improvement. At the one-month follow-up, complete resolution of pain was observed. At the two-year follow-up, no recurrent symptoms were detected. The previously described paresthesia had completely resolved, and no sensory disturbance was noted on physical examination (Figure 1).

Case 2

A 40-year-old male patient presented with chronic pain, burning sensation, and numbness localized to the third intermetatarsal space of the left foot. The patient indicated a distinct point of tenderness upon deep palpation in this area. His medical and occupational history was unremarkable. Although the patient had attempted conservative management by switching to non-constrictive footwear, he reported no relief from symptoms. A previous electromyography (EMG) was normal.

Clinical examination revealed tenderness localized to the third intermetatarsal space, with reproduction of symptoms upon deep palpation. Based on the typical localization and consistent clinical findings, Morton’s neuroma was suspected. Radiological imaging was not performed. Given the characteristic localization of symptoms and reproducible tenderness on examination, the clinical findings were considered sufficiently suggestive of Morton’s neuroma.

**Figure 2:**
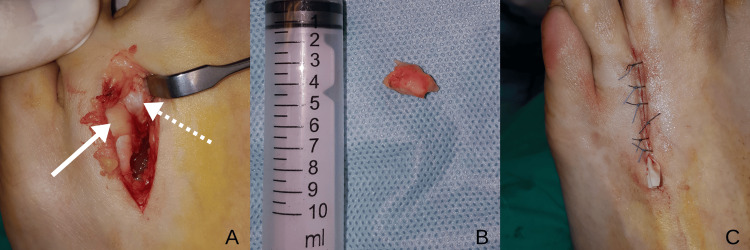
Intraoperative views of case 2 (A) Exposure of the third intermetatarsal space via dorsal approach. The solid arrow indicates the deep transverse metatarsal ligament; the dashed arrow indicates Morton’s neuroma. (B) Macroscopic appearance of the excised specimen. (C) Wound closure with Penrose drain placement.

The operation was performed under general anesthesia. Surgical excision of the neuroma was performed via a dorsal approach using a longitudinal incision. Sutures were removed on postoperative day 15. Postoperatively, the patient reported marked symptom improvement. At the one-month evaluation, complete pain resolution was observed. At the two-year follow-up, no recurrence was detected. The preoperative paresthesia had fully resolved, and no sensory deficits were noted on physical examination (Figure 2).

## Discussion

The disease is named after Thomas George Morton (1835-1903), a general surgeon who practiced in Pennsylvania [1]. Unlike the variable pain locations seen in various rheumatological disorders, pain due to Morton’s neuroma is specifically localized between the toes, presenting as metatarsalgia. The distribution of Morton’s neuroma is reported as 66% in the 3rd, 32% in the 2nd, and 2% in the 4th intermetatarsal spaces [4]. Even though the first intermetatarsal space is the widest, cases in this location have also been reported [6]. In 21% of patients, the neuroma is bilateral [4]. Although it is not as common as carpal tunnel syndrome, detailed statistics regarding its prevalence are available. It affects 87.5 per 100,000 women and 50.2 per 100,000 men in the European population [2].

Although the name of the disease is accepted as Morton’s neuroma in the literature, it is not actually a tumor of nerve tissue, but rather a benign perineural fibrosis of the common plantar digital nerve [3]. It occurs through the degeneration, demyelination, and sclerosis of nerve fibers [7]. Concentric layers of loose and fibrous connective tissue enclose the common plantar digital nerve, forming a protective tunnel during foot movements [8]. In the pathological process, as connective tissue thickens and becomes fibrotic, it begins to narrow the space available for the nerve. Patients describe the pain while walking as feeling like ‘walking on a pebble.’ While pain may subside with rest in the early stages of the disease, it becomes persistent in late stages of chronic cases. Various theories exist regarding the etiology of the disease. These are chronic trauma theory, entrapment theory, intermetatarsal bursitis theory and ischemic theory [5]. Additionally, mechanical compression of the nerve, particularly caused by wearing narrow or high-heeled shoes, is considered an important etiological factor [9,10]. Considering the professions and daily lives of our two patients, we were not able to detect any connection that could explain the development of this pathology.

Pain localized to the intermetatarsal space requires careful differential diagnosis. Conditions such as intermetatarsal bursitis, plantar plate injury, metatarsophalangeal joint synovitis, stress fractures of adjacent metatarsal heads, Freiberg disease, ganglion cysts, and other soft tissue masses may present with similar symptoms. In our cases, the characteristic localization of tenderness and reproduction of symptoms on deep palpation supported the diagnosis of Morton’s neuroma.

Radiological modalities have been increasingly utilized to support the diagnosis of Morton’s neuroma. Studies utilizing MRI have demonstrated a sensitivity of 93% and a specificity of 68%, whereas ultrasonography has shown 90% sensitivity and 88% specificity [11]. Although imaging may be particularly helpful in equivocal cases or when alternative pathologies are suspected, the diagnosis is frequently based on clinical findings in patients with a typical presentation. In our cases, the characteristic localization of pain and reproducible tenderness on examination were considered sufficient to proceed without additional imaging. Intraoperatively, the macroscopic appearance of the lesion was consistent with Morton’s neuroma. Although histopathological examination was not performed, the characteristic clinical presentation, intraoperative findings, and sustained postoperative symptom resolution supported the diagnosis.

While a pre-surgical conservative approach is mentioned in the literature, case series show that surgical excision provides satisfactory outcomes [4]. Therefore, we chose to proceed directly to surgical excision. Considering that our patients’ complaints have fully resolved and that more than two years have passed since the operation, we believe our choice of surgical approach to be justified. In his original description, Morton conceptualized the condition as a painful disorder of the metatarsophalangeal joint rather than a primary nerve pathology and therefore described a surgical procedure that included resection of the affected metatarsal head in order to relieve pressure on the involved nerve [9]. At the time, the interdigital nerve itself was not recognized as the principal source of symptoms. With the current understanding of the pathophysiology of Morton’s neuroma as an interdigital nerve entrapment, such bony resections are no longer considered necessary, and direct excision of the affected nerve is sufficient in most cases. Other surgical methods have been reported, including invasive distal metatarsal metaphyseal osteotomy and percutaneous release of the deep transverse metatarsal ligament [12].

We believe that the dorsal approach provides excellent visualization of the intermetatarsal space. The thinner skin on the dorsum of the foot should also be considered among the reasons for preferring this approach to reach the neuroma. A plantar incision requires dissection through thick skin and traversing the subcutaneous fat pad, and may necessitate dissection between the plantar flexor muscles. Additionally, due to the rich vascular supply of the plantar skin, this may increase the risk of bleeding complications. The dorsal approach is both straightforward and convenient. Rates of postoperative wound infections and wound healing problems are higher with the plantar approach compared to the dorsal approach [13]. Similarly, Nashi et al. demonstrated in a study of 52 patients that those treated with the dorsal approach required less time to return to work and experienced fewer complications compared to the plantar approach [14].

Although this case report includes a limited number of patients, the findings provide clinically relevant observations. Standardized pain scoring systems were not used, and radiological evaluation was not routinely performed. However, consistent clinical findings, intraoperative observations, and sustained long-term symptom resolution support the diagnosis and outcome.

## Conclusions

Morton’s neuroma should be considered in patients presenting with localized pain in the third intermetatarsal space, accompanied by characteristic tenderness on examination. In patients with typical clinical findings, careful physical examination may be sufficient to support the diagnosis. In our patients, surgical excision via a dorsal approach resulted in complete symptom resolution without recurrence or long-term sensory deficits during follow-up.

As Morton’s neuroma involves the common digital nerve, its surgical management requires meticulous dissection and precise nerve handling. Given their expertise in peripheral nerve surgery and detailed knowledge of nerve anatomy, plastic surgeons are well-positioned to contribute to the surgical treatment of Morton’s neuroma within a multidisciplinary setting.
